# Human Cord Blood and Bone Marrow CD34+ Cells Generate Macrophages That Support Erythroid Islands

**DOI:** 10.1371/journal.pone.0171096

**Published:** 2017-01-30

**Authors:** Eyayu Belay, Brian J. Hayes, C. Anthony Blau, Beverly Torok-Storb

**Affiliations:** 1 Clinical Research Division, Fred Hutchinson Cancer Research Center, Seattle, Washington, United States of America; 2 Department of Medicine, Division of Hematology, University of Washington, Seattle, Washington, United States of America; 3 Institute for Stem Cell and Regenerative Medicine, University of Washington, Seattle, Washington, United States of America; EFS, FRANCE

## Abstract

Recently, we developed a small molecule responsive hyperactive Mpl-based Cell Growth Switch (CGS) that drives erythropoiesis associated with macrophages in the absence of exogenous cytokines. Here, we compare the physical, cellular and molecular interaction between the macrophages and erythroid cells in CGS expanded CD34+ cells harvested from cord blood, marrow or G-CSF-mobilized peripheral blood. Results indicated that macrophage based erythroid islands could be generated from cord blood and marrow CD34+ cells but not from G-CSF-mobilized CD34+ cells. Additional studies suggest that the deficiency resides with the G-CSF-mobilized CD34+ derived monocytes. Gene expression and proteomics studies of the in vitro generated erythroid islands detected the expression of erythroblast macrophage protein (EMP), intercellular adhesion molecule 4 (ICAM-4), CD163 and DNASE2. 78% of the erythroblasts in contact with macrophages reached the pre reticulocyte orthochromatic stage of differentiation within 14 days of culture. The addition of conditioned medium from cultures of CD146+ marrow fibroblasts resulted in a 700-fold increase in total cell number and a 90-fold increase in erythroid cell number. This novel CD34+ cell derived erythroid island may serve as a platform to explore the molecular basis of red cell maturation and production under normal, stress and pathological conditions.

## Introduction

In vivo erythroid islands produce more than 2 million new reticulocytes every second in adult humans [1]. Despite the fact that the erythroid island was described a few decades ago as an erythropoietic niche consisting of a central “nurse cell” macrophage surrounded by maturing erythroblasts [2–4], our understanding of the interaction between macrophages and erythroblasts is still incomplete [5]. The majority of studies on erythroid islands were conducted either in vivo in rodents [6–9] or in long-term cultures of mouse cells [3, 10]. Mathematical models have also been explored to understand macrophage associated erythropoiesis [11, 12]. To date, there is no in vitro model for human erythroid islands that recapitulates the in vivo island.

In steady state erythropoiesis, the interaction of macrophages and erythroid cells (erythroid island) within the marrow microenvironment is critical for both early and late stages of erythropoiesis [13]. Erythroid progenitors in the bone marrow (BM) interact via multiple receptors, with multiple ligands on macrophages and matrix components within the marrow microenvironment [14]. Cell-cell interactions have been described and proven to be critical for island integrity serving as both positive and negative regulators of erythropoiesis [7]. The erythroblast macrophage protein (EMP) expressed on both erythroblasts and macrophages is the first ligand to be identified and found to be crucial for erythropoiesis [15]. As such, EMP null embryos die prenatally and show dramatic increase in the number of nucleated immature erythrocytes in the blood [16], indicating a disruption of the blood maturation steps. Other molecules that are involved in the macrophage erythroid interaction include vascular cell adhesion molecule-1 (VCAM-1) [16] and intracellular adhesion molecule-4 (ICAM-4) [17]. VCAM-1 is expressed on macrophages and interacts with α4β1 integrin on erythroblasts [17]. ICAM-4, expressed on the surface of erythroblasts, interacts with αV integrin on macrophages [17]. Secreted factors, TNF-α, TGF-β, IL-6 and IFN-γ are released by the central macrophage, and Gas-6, VEGF-A and PGF by erythroblasts [5, 18]. Macrophages also participate in the supply of iron, engulfing and digesting extruded nuclei expressing DNASE2 [8].

The erythroid island macrophage has also been implicated in red cell diseases. Recent reports suggest it contributes to the pathological progression of polycythemia Vera and β-thalassemia [19]. These studies reported macrophage depletion in mouse models of polycythemia Vera or β-thalassemia had beneficial effects in reversing key pathological features of both diseases. Chow and colleagues [20] also reported that macrophages appear to be essential for recovery from hemolytic anemia, acute blood loss, and myeloablation. These findings highlight the role of macrophages in stress and pathological erythropoiesis and their potential as targets for intervention.

Recently, we reported robust in vitro erythroid cell production and maturation from cord blood CD34+ cells without the addition of exogenous cytokines [21]. In these experiments, CD34+ cells were genetically engineered with Mpl-based Cell Growth Switch (CGS) comprised of a modified cytokine signaling domain (Mpl) linked to an artificial dimerization domain. Addition of a small drug molecule called a chemical inducer of dimerization (CID) then triggers dimerization and signaling through the CGS [22]. The recently developed hyperactive version of the CGS expands engineered cord blood CD34+ cells up to 100-fold mainly into the erythroid lineage in the absence of added erythropoietin (EPO) [21].

In this study, scanning electron and light microscopy of CGS expanded CD34+ cells revealed maturing erythroblasts arranged in a ring around a central macrophage, a markedly close two-dimensional representation of the classic three-dimensional erythroid island morphology [23]. The possibility that these represented functional erythroid islands was supported by the expression of erythroid macrophage interaction molecules such as EMP and ICAM-4 and the detection of secreted erythroid island niche-associated factors. Transcriptome analysis of the macrophage showed the expression of cell surface and intracellular factors known to be involved in in vivo erythroid-macrophage interaction. The erythroid island associated erythropoiesis leads to the erythroid specification of cord blood CD34+ cells with maturation into orthochromatic normoblasts, the most mature nucleated stage of erythroid differentiation. When conditioned medium from cultures of CD146+ marrow fibroblasts was added to the assay erythropoiesis increased enucleation and engulfment of pyrenocytes could be observed. Similar results were obtained with marrow CD34+ cells but not with CD34+ cells harvested from G-CSF-mobilized blood.

## Methods

### Cell Growth Switch Lentiviral vectors

The hyperactive CGS self-inactivating Lentiviral vector used in these studies has been described previously [21]. In brief, the hyperactive CGS receptor cassette is transcribed from the constitutively active Murine Stem Cell Virus (MSCV) promoter and is comprised of a hybrid sequence encoding the modified binding domain FKBP (F36V) linked to a cDNA encoding the intracellular domain of mouse Mpl with Y582F hyperactivating mutation. The constitutively active PGK promoter drives the GFP cassette which serves as a marker for CGS transduction. Lentiviral vectors were generated by co-transfection of 293T cells and concentrated 100-fold before use and tittered at the viral production resource at the Fred Hutch.

### Primary cell isolation, culture and transduction

All tissue were collected and experiments conducted according to guidelines approved by the Fred Hutch Institutional Review Board. Human cord blood was obtained from the Bloodworks NW (Seattle, WA). Cord blood mononuclear cells were enriched by Ficoll density gradient centrifugation. CD34+ cells, cord blood monocytes, BM monocytes and cultured erythroid cells were enriched by immunomagnetic separation using CD34, CD14 and CD235a microbeads respectively (Miltenyi Biotech). Purity of enriched cells was 90+/-5% in cord blood CD34+, 98+/- 1% in CD14+ monocytes and 99% in CGS expanded CD235a+ erythroid cells. Anonymized adult BM and G-CSF-mobilized CD34+ cells (purity of >98%) were obtained from the cell processing core at the Fred Hutch following donor consent. For CGS viral transduction, CD34+ cells were pre-stimulated in IMDM supplemented with 10% FBS (IMDM/FBS), 50 ng/ml rhSCF, 20 ng/ml rhIL-6, 20 ng/ml rhTPO and 100 ng/ml rhFlt3L and incubated at 37°C in 5% CO_2_. All cytokines were purchased from PeproTech (Rocky Hill, NJ). After 6–9 hrs of pre-stimulation, 5 μg/ml polybrene was added and cells were transduced overnight with CGS at an m.o.i of 20. Cells were washed and replated in IMDM/FBS plus 100 nM AP20187 without the addition of exogenous cytokines. At specified days cells were collected, washed and either saved for analysis or re-plated in fresh medium at a target concentration of 5x10^5^ cells/ml.

### Stromal cell lines and preparation of conditioned medium (CM)

Human CD146+ (HS27a; ATCC CRL-2496) and CD146- (HS5; ATCC CRL-11882) BM stromal cell lines [24] were grown in 150 mm dishes at 2x10^6^ cells/ml in RPMI-1640 supplemented with 10% FBS. Conditioned medium was harvested after 3 days, clarified by centrifugation and concentrated ten-fold using Centriprep YM-10 Centrifugal Filters (EMD Millipore) and stored at -20°C. The CM was diluted with three volumes of IMDM/FBS before use. To prepare the control cord blood CD34+ CGS culture supernatant, 2x10^6^ cells/ml from day 11 CGS expansion culture was harvested and re-plated further for 3 days and supernatant collected.

### Cell analysis

Cells were harvested for analysis, after washing twice in 1%FBS in PBS supplemented with 2 mM EDTA. Cells were stained with fluorochrome-conjugated antibodies CD34-PECy7a, CD169-PE, CD14-PE, CD106-APC, CD11C-APC, CD115-PE, CD235a-APC (BD Biosciences), CD233-PE, CD49d-APC (Militeny Biotec) and CD206-PECy7a (eBioscience). Flow cytometry analysis was done on a FACSCanto 6-color flow (BD Biosciences) and data collected from 30,000 events and analyzed using the Flow Jo software. Sorting of CD206+ macrophages from CGS culture was performed on a FACSAria cell sorter (BD Biosciences). Cytospins were prepared by suspending cells in 200 μl 2% FBS/PBS, and centrifuged onto slides at 72 g for 5 min, air-dried, stained with Wright-Giemsa and imaged at 40x (Evos XL, Life technologies). Florescence images were taken using Nikon Eclips Ti microscope at 40x objective.

### Colony-forming unit (CFU) assay and time lapse microscopy

Cord blood derived CD34+ cells transduced with the CGS were assessed for CFU by plating 40K cells in cytokine free MethoCult^™^ H4230 (STEMCELL Technologies) supplemented with 100 nM/ml AP20187. Plates were incubated at 37°C, 5% CO_2_ for 14 days and individual colonies were imaged, picked manually, washed, cytospun and stained with Wright-Giemsa. For time lapse microscopy, BM CD34+ cells were transduced with CGS and expanded for three days, 200K cells were further plated in cytokine free MethoCult^™^ H4230 (20% cell suspension in 80% MethoCult with 100 nM/ml AP20187). GFP+ (CGS expressing cells) were imaged at day 7 using Nikon Eclipse Ti-E (ISCRM, University of Washington). For morphological analysis, live imaged cells were harvested form the MethoCult, spun onto glass slides and then stained with Wright-Giemsa.

### ELISA

Proteins in conditioned media were measured in triplicate using Luminex microbeads technology (Luminex^®^ 200^™^ System, Life technologies) at the Immune monitoring laboratory of the shared resources at the Fred Hutch.

### Scanning Electron Microscopy (SEM)

Cell Growth Switch expanded cord blood CD34+ cells were harvested at day 11 of culture, washed and replated on glass cover slips in a 24-well plate in IMDM/FBS supplemented with 100 nM AP20187. After 3 days of culture cells were fixed *in situ* using 2.5% glutaraldehyde/ 2% paraformaldehyde buffer at 37°C. Fixed cells were processed for Scanning electron microscopy at the Electron Microscopy shared resource at the Fred Hutch and images were captured with a JEOL 5800 electron microscope (JEOL, Tokyo, Japan).

### Microarray hybridization and data analysis

Microarray hybridization and data analysis were conducted at the Fred Hutch Genomics Shared Resource. In brief, after 14 days of CGS expansion of cord blood CD34+ cells, macrophages were flow sorted based on CD206 expression (BD Bioscience). Control monocytes were isolated from unmanipulated cord blood mononuclear cell using CD14 microbeads (Miltenyi Biotec‎). Total RNA was extracted using RNeasy spin columns (Qiagen). cDNA synthesis and hybridization to Illumina HumanHT-12 v4 Expression BeadChips (Illumina) were done following the manufacturer’s standard protocols. Microarray data was assessed for quality and quantile normalized using the Bioconductor package lumi [25]. Initial filtering included flagging probes that were below a signal ‘‘noise floor”, which was calculated as the 75^th^ percentile of the negative control probe signals within each array.

## Results

### In vitro CD34+ cell derived erythroid islands

**Fig 1:**
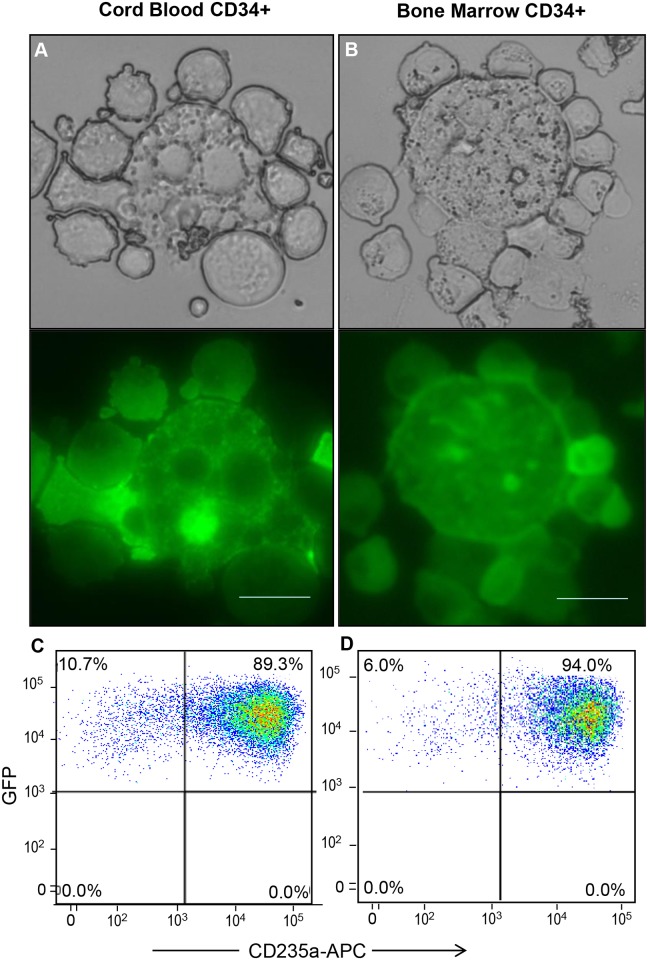
CD34+ cells derived in vitro erythroid islands. Cord blood and bone marrow CD34+ cells were transduced with Lentiviral vectors encoding the cell growth switch (CGS) and expanded in IMDM/10% FBS in the presence of 100 nM AP20187. At day 14 cells were collected gently, spun onto glass slides and imaged by bright filed and fluorescence microscopy. (A) Cord blood and (B) bone marrow CD34+ cell derived erythroid islands are shown, an aliquot of the collected cells was treated with EDTA to prepare a single cell population for flow analysis. Representative flow cytometry profiles demonstrating the CGS expanded (GFP+) and erythroid (CD235a) expression pattern of (C) cord blood and (D) bone marrow CD34+ cells is presented (Scale bar 50 μm).

**Fig 2:**
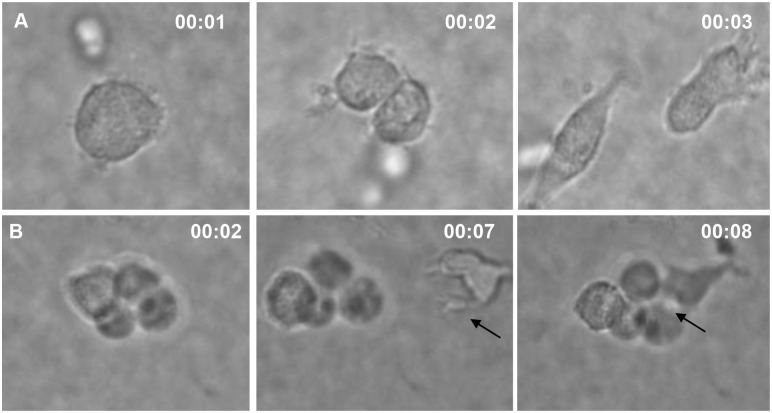
Representative snapshots showing a macrophage actively dividing and joining a tetrad of erythroblasts. (A) Time lapse sequence showing macrophage actively dividing in CGS culture without the addition of exogenous cytokine and (B) a macrophage actively joining a tetrad of CGS expanded erythroblasts.

We recently developed a hyperactive CGS that drives macrophage associated cytokine free erythropoiesis [21]. To explore the in vitro erythroid macrophage interaction we transduced cord blood and adult BM derived CD34+ cells with Lentiviral vector encoding the hyperactive CGS and expanded cells in the presence of 100 nM AP20187 without the addition of exogenous cytokines. After 14 days of expansion cells were harvested and examined by microscopy and flow cytometry. Erythroid cells arranged in a ring around a central macrophage were detected in both CGS-expanded (GFP+) cord blood (Fig 1A) and adult BM CD34+ cells (Fig 1B) mimicking the structure of the BM erythroid islands [2]. Cells from the same culture were further analyzed by flow cytometry. We found that 89.3% of CGS expanded cord blood (Fig 1C) and 94% of GFP+ adult BM CD34+ (Fig 1D) cells express glycophorin A (CD235a) indicating that the GFP+ cells surrounding the central macrophages were indeed erythroid cells. The erythroid islands start to appear within three days of CGS culture (S1A Fig) in all experiments we conducted from different donors of CD34+ cells. Each central macrophage was surrounded by 4 to 9 erythroblasts (S1B Fig) and on average we found 30 erythroblasts for every macrophage at day 14 of culture. Phenotypically the macrophages were ITGAX, CD206+, CD14+, CD169low, CD106low and CD115- (S2 Fig). When CGS transduced cord blood CD34+ cells were seeded into cytokine free semi-solid media at very low density, all the 20 colonies picked and analyzed contained macrophages (S3 Fig) suggesting a common progenitor for both the erythroblasts and the central macrophages. However, time lapse imaging showed isolated macrophages dividing (Fig 2A) and some moving considerable distance to join a 4-cell culture of GFP+ erythroblasts (Fig 2B). This would suggest the macrophages and erythroblasts could be derived from different progenies contained in the CD34+ cells population.

### In vitro erythroid island associated terminal erythroid differentiation

**Fig 3:**
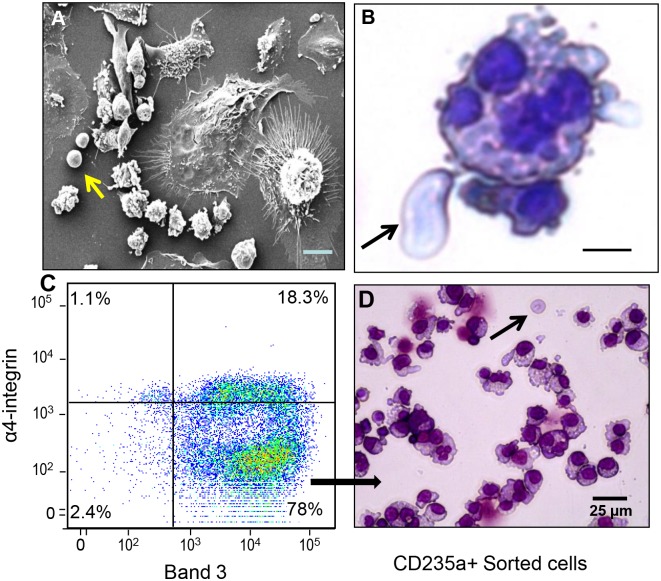
Cell growth switch expansion of cord blood CD34+ cells lead to terminal erythroid differentiation. Cord blood CD34+ cells were expanded with the CGS in the presence of 100 nM AP20187. At day 14 cells were fixed in suite and electron microscopic studies revealed (A) a patterned direct association of erythroblasts around a central macrophage with processes extended towards erythroblasts, (B) Wright-Geimsa stained slides show a macrophage with multiple engulfed nuclei and enucleated red blood cell (see arrows) (40 x objective). (C) Flow cytometry analysis show α4-Integrin (low) and Band 3 (high) erythroid cells typical of the orthochromatic stage of erythroid differentiation. (D) Morphology of flow sorted (CD235a+), Wright-Geimsa stained orthochromatic normoblasts with minimal enucleation (arrow) is presented (Scale bar 50 μm).

To identify the stage of erythroid differentiation in the CGS expanded cord blood CD34+ cells and further confirm *in situ* island formation, we conducted morphological studies of *in situ* fixed day 14 culture using scanning EM and the conventional Wright-Giemsa staining and flow analysis. Scanning EM studies revealed a patterned direct association of erythroblasts around a central macrophage with processes extended towards erythroblasts (Fig 3A). The Wright-Giemsa morphological study also revealed complete enucleation and red cell release although rare (Fig 3B). To identify the maturation stage of erythroid island associated cells we used a flow cytometry-based staging of erythroid differentiation as determined by Band 3 and α4-integrin expression [26]. As erythroblasts mature, the expression of α4-integrin decreases while that of band 3 increases progressively. Day 14 CGS expanded cells were harvested and stained for band 3 and α4-integrin. As shown in Fig 3C, 78% of the CGS expanded GFP+ cells (>90% of GFP+ cells are CD235a+) were band 3 high and α4-integrin low indicative of the pre-reticulocyte orthochromatic stage of erythroid differentiation. This finding was further confirmed by Wright-Giemsa staining and morphological study of the CD235a+ sorted erythroid cells (Fig 3D). Orthochromatic normoblasts with a decreased nuclear-cytoplasm ratio, condensed nuclei pushed to the edge of the cells with minimal enucleation were detected (Fig 3D arrow).

### Macrophage erythroblast interaction is mutual

**Fig 4:**
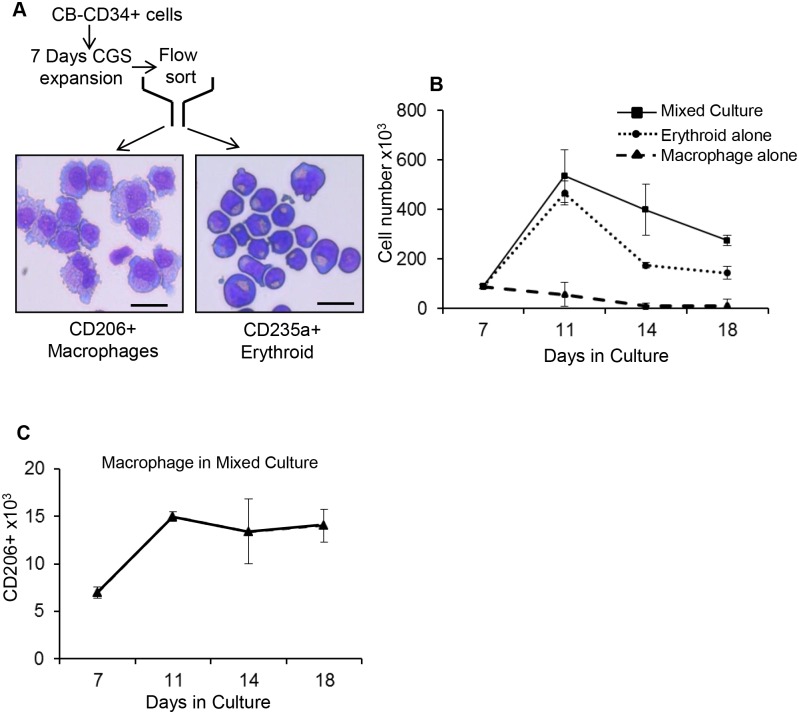
Erythroid macrophage co-cultures yield maximum expansion. Cord blood CD34+ cells were expanded with the CGS in the presence of 100 nM AP20187. (A) At day 7 of culture erythroid cells and macrophages were flow sorted based on CD235a and CD206 expression respectively. Morphology of sorted erythroid and macrophages was confirmed by cytospin and Wright-Giemsa staining. (B) Sorted erythroid and macrophages were cultured as erythroid alone macrophage alone and mixed (9 part erythroid and 1 part macrophage), at equal cell total densities. (C) Total macrophage number was monitored over time in mixed culture based on CD206 expression (Scale bar 25 μm).

**Fig 5:**
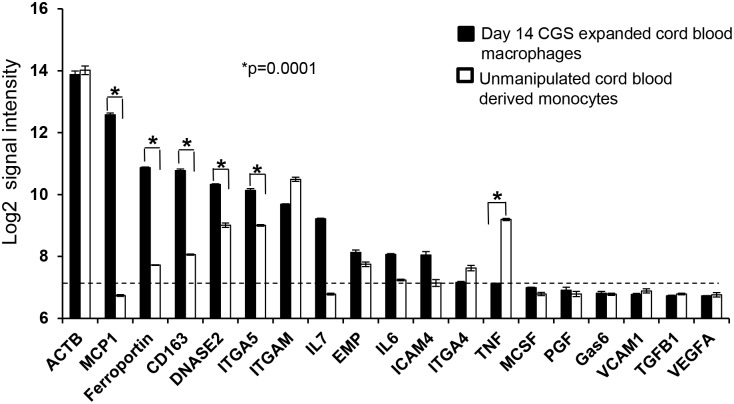
Erythroid island associated gene expression. Macrophages from day 14 CGS expanded cord blood CD34+ cells culture were flow sorted based on CD206 expression and control unmanipulated cord blood monocytes separated by CD14 immunomagnetic beads. Whole genome transcriptome analysis was conducted using Illumina HumanHT-12 v4 Expression assay. Selected genes are presented that are known to be associated with erythroid island niche. The 75th percentile of the negative control probes was used to define the cutoff value of log 7 signal intensity. Genes that are at or below this level are in the 'noise'. The ACTB house keeping gene is included as a reference and the average of three experiments is presented.

To explore whether the macrophages and erythroid cells are interdependent, we flow sorted day 7 CGS expanded cord blood CD34+ cells into CD206+ macrophages and CD235a+ erythroid cells (Fig 4A). The isolated cells were cultured separately or mixed (90% erythroid and 10% macrophage). By day 18 of culture, erythroblasts co-cultured with macrophages increased in number 3.7-fold while the erythroblasts cultured alone show a 1.6-fold increase. Macrophages in mixed culture show a 2.7-fold increase but those cultured alone die off (Fig 4B). Based on the percentage of CD235a+ and CD206+ cells in the mixed culture, we calculated the absolute number of macrophages and erythroid cells and found that both macrophages and erythroid cells increased. This suggested an association between the two cells was required for expansion in the CGS culture condition. As mentioned above, live imaging of CGS expansion of BM CD34+ cells revealed actively dividing macrophage and a macrophage being attracted to a dividing erythroblast. Morphologic study of cytospins from cell harvested post live imaging revealed erythroblast and macrophage forming erythroid islands (S4A Fig). We also observed erythroblasts in karyokinesis within the erythroid islands (S4B and S4C Fig). To assess the molecular basis of the macrophage erythroid interaction, CD206+ macrophages were flow sorted from day 14 CGS expanded cord blood CD34+ cells for gene expression studies. The level of gene expression of known erythroid island associated genes CD163, ICAM-4, DNASE2, ITGAM and ferroportin were significantly (p = 0.0001) increased in CGS derived macrophages compared to unmanipulated cord blood derived monocytes. Macrophage chemoattractant protein 1 (MCP1) was highly expressed along with other cytokines and chemokines (Fig 5). In addition, we compared the gene expression pattern of CGS erythroid island associated macrophages to that of BM derived CD14+ cells from healthy donor by qPCR. We found a three to seven fold upregulation of erythroid island associated genes ferroportin, DNASE2 and ICAM-4 compared to BM derived CD14+ cells (S5 Fig).

### G-CSF-mobilized CD34+ cells don’t form in vitro erythroid islands

**Fig 6:**
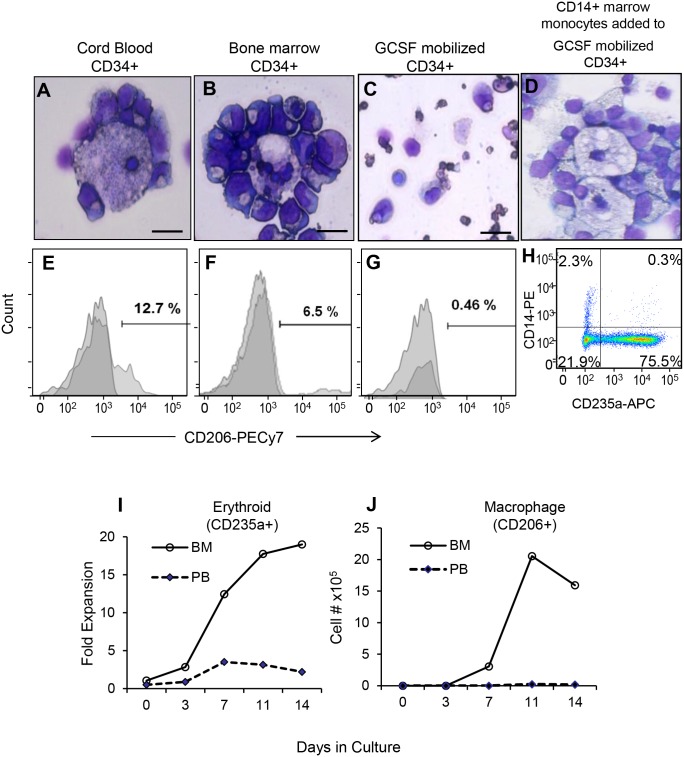
Cell Growth Switch transduced G-CSF-mobilized CD34+ cells do not form in vitro erythroid islands. Cord blood, bone marrow and G-CSF-mobilized CD34+ cells were expanded using the CGS in the presence of 100 nM AP20187. CD14 monocytes from health donor was added to the G-CSF-mobilized culture. Representative Wright-Giemsa stained images of (A) cord blood CD34+ cells, (B) bone marrow CD34+ cells and G-CSF-mobilized peripheral blood CD34+ cells (C) without exogenous monocytes and (D) with addition of 15% marrow derived monocytes are shown. The corresponding flow cytometric histograms demonstrate immunofluorescence staining pattern of the macrophage marker CD206 in (E) cord blood (F) bone marrow and (G) G-CSF-mobilized CD34+ cells. Comparisons of fold expansion of (H) erythroid (CD235a+) and (I) absolute macrophage (CD206+) cell number in bone marrow CD34+ and G-CSF-mobilized CD34+ cells is presented. Fold expansion is relative to starting cell number and data is from two independent donors (scale bar 25 μm).

We have reported the expansion of G-CSF-mobilized CD34+ cells using the hyperactive CGS to be inefficient compared to cord blood derived CD34+ cells [21]. We hypothesize that the inefficiency of expansion and rapid decline of CGS expanded G-CSF-mobilized CD34+ cells could be attributed to difference in macrophage function. We compared erythroid islands formation and erythroid expansion in CGS-based cultures of cells from cord blood, BM and G-CSF- mobilized CD34+ cells. We harvested cells from day 7 expansion cultures and conducted morphological and flow based studies. Unlike the formation of in vitro erythroid islands in CGS expanded cord blood CD34+ (Fig 6A) and BM CD34+ (Fig 6B) cells, we did not observe any erythroid island formation from G-CSF-mobilized CD34+ cells (Fig 6C). Subsequent flow cytometry analysis demonstrated that cord blood CD34+ cells (Fig 6E) and BM CD34+ (Fig 6F) culture generate 12.7 and 6.5% CD206+ macrophages respectively while the G-CSF-mobilized CD34+ culture show only 0.5% CD206+ macrophages (Fig 6G). Comparing the fold expansion of CD235a+ erythroid cells in BM and G-CSF-mobilized CD34+ cells, we observed that CGS expanded BM-CD34+ cells lead to a 10 fold increase in erythroid cells compared to mobilized CD34+ cells (Fig 6I). Interestingly, the total number of CD206+ macrophages increased in CGS expanded BM-CD34+ cells while G-CSF-mobilized CD34+ cells failed to do so (Fig 6J). Further characterization of CGS expansion culture from the three CD34+ cell sources revealed a faster rate of CID selection as measured by GFP expression and corresponding erythroid commitment (CD235a+) in BM and G-CSF-mobilized CD34+ cells. The CGS expansion of cord blood derived CD34+ cells show increased percentage of CD206+ macrophages compared to the BM and G-CSF-mobilized CD34+ cells which partly explain why CGS expansion of cord blood CD34+ cells leads to increased fold expansion and survival of erythroid cells in culture.

### G-CSF-mobilized CD34+ cells form erythroid islands when supplemented with marrow monocytes

The deficiency in the formation of in vitro erythroid island in G-CSF-mobilized CD34+ was rescued by the addition of bone marrow monocytes. We co-cultured bone marrow derived CD14+ monocytes from healthy donors with G-CSF-mobilized CD34+ cells (15% CD14+ monocytes and 85% G-CSF-mobilized CD34+ cells) and transduce the mix with the CGS lentiviral vectors and start expansion in the presence of 100 nM AP20187. Interestingly, the CGS expansion of G-CSF-mobilized CD34+ culture supplemented with bone marrow monocytes form erythroid islands (Fig 6D); showed enhanced proliferation of cells in to the erythroid linage (Fig 6H) and improved cell survival up to three weeks (data not shown). The control culture of G-CSF-mobilized CD34+ cell without the addition of bone marrow monocytes failed to form erythroid islands and die within 10 days.

### Bone marrow stromal cell conditioned medium enhance erythroid differentiation

**Fig 7:**
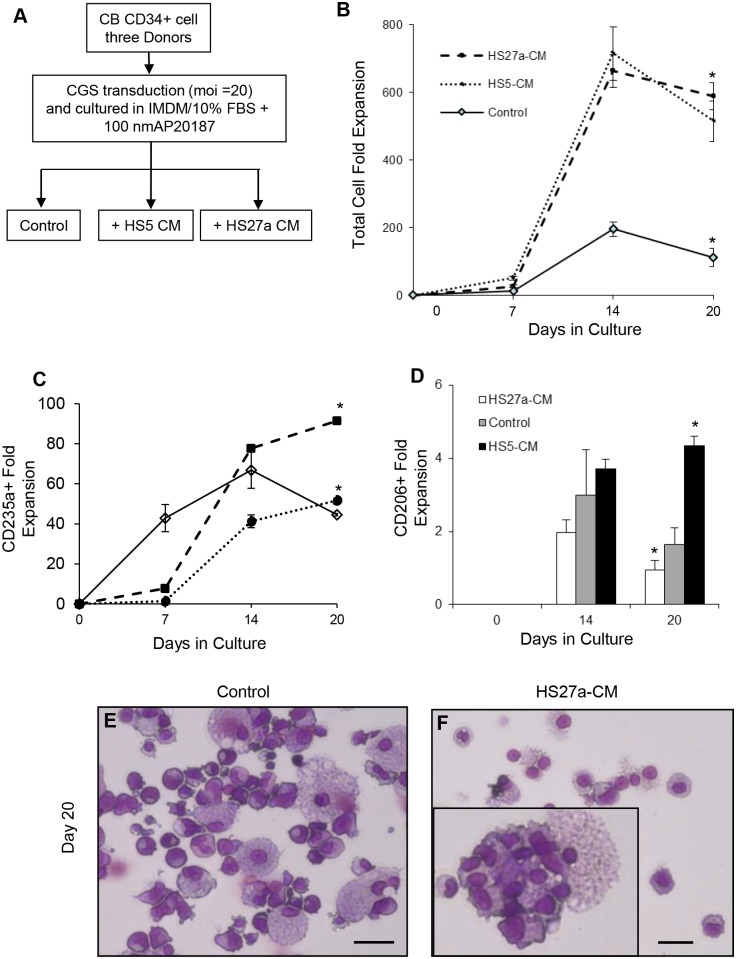
Bone marrow fibroblast conditioned medium enhance island associated erythropoiesis. (A) Schematics of experimental design to test the effect of CD146+ (HS27a) and CD146- (HS5) bone marrow fibroblast conditioned medium on CGS based expansion of cord blood CD34+ cells. Cell aliquots were harvested at the indicated time points counted and analyzed by flow cytometry and are reported as fold-expansion based on the starting cell number. The fold change of (B) total, (C) erythroid (CD235a+) and (D) macrophage (CD206+) cell number in HS27a, HS5 and control no conditioned medium treatment is presented. Representative images of Wright-Giemsa stained cytospins from day 20 expansion products are shown in panels (E) control and (F) with HS27a-CM treatment. Inset in panel (F) indicate a macrophage with multiple engulfed nuclei. Data represent the mean ± SE from three independent experiments using three cord blood donors. *P* values are based on the *t* test (scale bar 25 μm).

**Fig 8:**
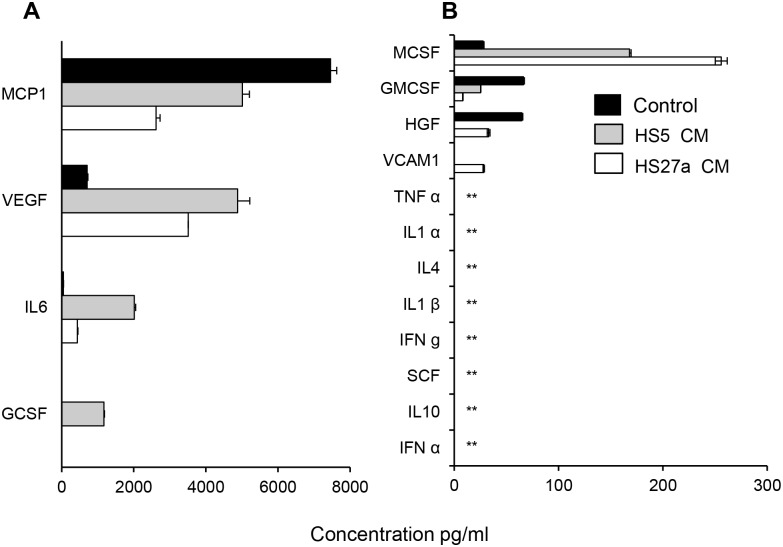
Quantification of secreted proteins. The level of secreted proteins in culture supernatant was measured by enzyme-linked immunosorbent assay. Proteins secreted at (A) 300–8000 pg/ml and (B) lower than 300 pg/ml are indicated. The control is a culture supernatant from CGS expanded cord blood CD34+ cells alone. Conditioned medium was harvested from a starting cell number of 2 x10^6^/ml HS5 and HS27a marrow fibroblast. Mean of three independent measurements are presented. ** less than the lowest detection limit of the assay.

Conditioned medium from cloned stromal fibroblast HS27a and HS5 [24] have been reported to induce a tissue macrophage and pre-dendritic cell phenotypes in monocytes respectively [27]. Addition of HS27a and HS5 CM to our CGS culture (Fig 7A), resulted in up to 700 and 662 fold increase in total cell number in HS27a and HS5 CM treated cells respectively. The control with no CM lead to a 196 fold increase (Fig 7B). Tracking the fold change in erythroid cells (CD235a+), we observed that the HS27a CM enhanced erythroid commitment by 90 fold compared to the 51 fold increase in the control culture while the HS5 CM treatment lead to a significant delay in erythroid commitment (Fig 7C). Since our CGS based expansion is associated with macrophages, we also compared the fold change in macrophage number and observed that the HS5 CM show slight increase in macrophage number (CD206+) compared to the HS27a CM treated and the control (Fig 7D) with distinct morphology (S6 Fig). Surprisingly, at day 20 of culture, the HS27a CM treatment show engulfment of extruded nuclei by macrophages (Fig 7F insert) compared to the untreated control (Fig 7E). Analysis of secreted proteins in the CM from CGS expanded cord blood CD34+ cells, HS5 and HS27a fibroblasts demonstrated that macrophage chemoattractant protein1 (MCP1), vascular endothelial growth factor (VEGF) and IL-6 being the major proteins secreted by the three conditions (Fig 8A). The cytokines IL-1, IL4, IL-10, TNFα, SCF and IFN α were undetectable (Fig 8B). Interestingly, we found that G-CSF and VCAM-1 were secreted exclusively by the HS5 and HS27a fibroblasts respectively (Fig 8).

### Recombinant VCAM-1 enhance Cell Growth Switch based erythroid expansion

It has been reported that VCAM-1 is expressed on macrophages and interacts with α4β1 integrin on erythroblasts enhancing macrophage-erythroid interaction [17]. We detected VCAM-1 in the conditioned medium from HS27a fibroiblasts that was associated with further enhancement of erythroid expansion (Fig 7C). We hypothesized that the addition of recombinant human VCAM-1 (rhVCAM-1) may augment erythroid proliferation. Cord blood CD34+ cells were expanded in the presence of 100 nM AP20187 alone, 50 ng/ml rhVCAM-1 and HS27a CM. We found that addition of HS27a CM and rhVCAM-1 resulted in a 666 and 422 fold increase in erythroid cell number respectively while the AP20187 alone shows a 202 fold increase relative to the day three CD235a+ cells (S7 Fig). This supports the role of VCAM-1 in HS27a conditioned medium in the erythroid commitment of CD34+ cells. However, rhVCAM-1 alone was not as efficient as the HS27a conditioned medium indicating other soluble factors present in the HS27a CM contribute in the CGS induced erythroid commitment and expansion of cord blood CD34+ cells.

## Discussion

We have identified and characterized in vitro erythroid islands that support erythropoiesis without the addition of exogenous cytokines from cord blood and BM but not G-CSF-mobilized CD34+ cells. Within two weeks of culture the in vitro erythroid island supported erythropoiesis generates up to 80% orthochromatic normoblasts. The number of normoblasts produced could be increased by the addition of a specific BM fibroblast conditioned medium which also allowed for detectable enucleation.

Our knowledge of the molecular mechanisms, signaling pathways, and genes involved in the late stage macrophage associated erythropoiesis is limited by our inability to mimic the in vivo erythroid island. The majority of studies on erythroid islands are conducted in rodents [6–9] and produce results that cannot be extrapolated to humans [28]. This limits our understanding of terminal erythroid maturation and is a barrier in the development of treatments for anemias associated with defects in terminal stages of erythropoiesis [29]. Since enucleation of erythroblasts is the major bottleneck in the production of stem cell derived red blood cells in vitro [30], the development of an in vitro model for human erythroid islands that allows for terminal differentiation could serve as a platform for drug screening and the study of normal versus pathological erythropoiesis.

Scanning electron and light microscopic studies of cord blood and BM CD34+ cells expanded in vitro revealed erythroid cells in contact with cytoplasmic processes emanating from a central macrophage that approximates the structure of the in vivo erythroid islands [2]. In vitro erythroid islands have been reported in long-term cultures of mouse BM [3] and murine differentiating embryonic stem cells [10]. Our study is the first to characterize in vitro human CD34+ cell derived erythroid islands under cytokine free conditions. Morphologically the erythroblastic island macrophages are very large with diameters frequently exceeding 30 μm and have a nuclear/cytoplasmic ratio smaller than what has been previously described [13]. Phenotypically the erythroid island macrophages were CD206+, CD11C+, CD14+, CD169low, CD106low and CD115-. The macrophages appear as early as 3 days of culture and were detected in every hemoglobinized colony (S1 Fig) suggesting the two cells are co-dependent. The erythroblasts were predominantly (78%) orthochromatic normoblasts (Fig 3C) similar to those found in the sinusoid of the BM [13]. Both the macrophage and erythroid cells appear to benefit from the interaction, in mixed culture, we observed a 3.7 fold increase in erythroid cell number relative to input cells while the erythroid only culture lead to a 1.6 fold increase (Fig 4). This is in agreement with the study of Rhodes et al [31] who reported that erythroid cells co-cultured with macrophages show a 3-fold increase in proliferation. Macrophages cultured alone decline in cell number and die off but those in co-culture show a 2.7-fold increase. Live imaging of CGS expanded BM CD34+ cells in a semi-solid medium further support macrophage and erythroblast interactions and active cell division.

The in vitro erythroid island identified in our CGS-expanded cord blood CD34+ cultures share several properties with the in vivo erythroid island. We detected EMP, ICAM-4 and low level of VCAM-1 reported to be crucial for island integrity [17, 32, 33]. Gene expression study also revealed a significant (p = 0.0001) increase in the expression of MCP-1, CD163, the iron transport proteins ferroportin and DNASE2 in our in vitro generated erythroid island macrophages compared to unmanipulated cord blood monocytes (Fig 5). CD163 is a known macrophage surface glycoprotein receptor for erythroblast adhesion [34]. Within the erythroid island, ferroportin and DNASE2 channel iron and degrade extruded nuclei respectively critical steps in normal erythropoiesis [5, 35]. In the present study, we detected higher level of MCP-1 both at the mRNA and protein level and its role in erythropoiesis and erythroid island formation needs further studies. The angiogenic factors, vascular endothelial growth factor (VEGF) and placental growth factor (PGF) that promote erythroblasts-macrophages interaction within the erythroid island [18, 33] were detected in the culture supernatant.

Interestingly, G-CSF-mobilized CD34+ cells failed to form in vitro islands (Fig 6C) unlike cord blood and BM derived CD34+ cells in vitro. When supplemented with marrow monocytes, G-CSF-mobilized CD34+ cells form erythroid islands and show improved erythroid commitment (Fig 6D and 6H) and survival. This could be associated with the inherent molecular difference between G-CSF-mobilized and marrow derived CD34+ cells as we reported previously [36], the effect of G-CSF mobilization on erythropoiesis [37] and/or the phenotype of G-CSF-mobilized CD34+ cell derived macrophages. This necessitate further studies to identify both the erythroid commitment potential and nature of monocytes derived from different CD34+ cells sources.

Erythroid island macrophages arise from resident monocyte precursors [13]. Conditioned medium from HS27a and HS5 cells have been shown to induce monocytes to a macrophage and pre-dendritic phenotype respectively [27]. We observed that the HS27a CM enhanced erythroid commitment, engulfment of extrude nuclei and enucleation (Fig 7). This associates with the acquisition of the macrophage phenotype and secretion of VCAM-1. When HS5 CM was added to CGS culture, erythroid expansion was decreased and delayed. This may be explained in part by the fact that HS5 fibroblast unlike HS27a secretes G-CSF (Fig 8A) and G-CSF has been reported to block erythropoiesis by depleting erythroid island macrophages [37].

In a cytokine free, small molecule based strategy, the CGS leads to efficient erythroid commitment of HSCs into the orthochromatic normoblasts stage (~80%). However, the enucleation step to generate red blood cells is very minimal. Generation of red blood cell from HSC has been achieved in vitro in liquid culture containing a cocktail of cytokines and growth factors [38, 39]. Proof of principle studies also demonstrated the function of in vitro generated red blood cells in vivo [40]. These strategies require different combinations of cytokines including EPO, VEGF and IL-3 in a four step passage protocol. CGS generated orthochromatic normoblasts could be tested for enucleation by transferring into the last enucleation step of liquid culture. The effect of different cytokines, especially EPO in the enucleation step of CGS generated orthochromatic normoblasts should be further explored. The efficiency of current in vitro red cell production is still not comparable to that of the bone marrow. The rate limiting step being terminal erythroid commitment and enucleation. To overcome this hurdle, we need to understand the molecular bottlenecks in the enucleation process within the context of the BM microenvironment. Comparison of the molecular signature of orthochromatic normoblasts derived from the bone marrow to those generated in vitro at a single cell level by RNA-Seq has the potential to identify key players in the enucleation process.

In summary, we have identified and characterized erythroid islands that develop from cord blood or marrow CD34+ cells but not in G-CSF-mobilized CD34+ cells. The in vitro erythroid islands have similar morphology, cell-cell interaction molecules, cytokine and growth factor expression characteristic of in vivo erythroid islands. This novel model of erythroid islands may be used as a platform to explore the molecular basis of erythroid island function in normal, stress, and pathological erythropoiesis. Although the in vitro erythroid island associated erythroid differentiation of CD34+ cell reach the pre-reticulocyte orthochromatic normoblast stage, enucleation is still rare. Further studies are required to identify the molecular bottlenecks in the in vitro enucleation processes to fully recapitulate the efficiency of in vivo erythropoiesis.

## Supporting Information

S1 Fig Appearance of erythroid islands. Cord blood CD34+ cells were transduced with Lentiviral vector encoding the CGS and plated in IMDM/FBS supplemented with 100 nM AP20187. Cells were harvested at the indicated time points, cytospun and stained with Wright- Giemsa. (A) Representative images of erythroblast and erythroid islands in culture and (B) Average number of cells surrounding a central macrophage. The mean number of erythroblast around a macrophage in 20 randomly selected islands is presented. (scale bar 25 μm).
(PDF)

S2 Fig Phenotype of macrophages in expanded culture of cord blood CD34+ cells. Cord blood CD34+ cells were transduced with Lentiviral vector encoding the CGS and plated in IMDM/FBS supplemented with 100 nM AP20187. At day 14 of culture cells were harvested stained with CD206 antibody and flow sorted. Sorted macrophages were stained with the indicated cell surface markers and flow analyzed, representative flow cytometric histograms are presented.
(PDF)

S3 Fig Central macrophages in individual colonies. Cord blood CD34+ cells were transduced with Lentiviral vector encoding the CGS and plated in a cytokine free MethoCult semi solid medium supplemented with 100 nM AP20187. At day 14, red colonies were manually picked and Wright-Giemsa stained. Representative images of individual colonies and the corresponding central macrophage from each colony are presented (n = 20 colonies, scale bar 25 μm).
(PDF)

S4 Fig Erythroblasts and macrophages from live video images. Bone marrow CD34+ cells were transduced with Lentiviral vector encoding the CGS, and plated in a cytokine free MethoCult semi solid medium supplemented with 100 nM AP20187. After collection of time lapse images cells were harvested, cytospun and Wright-Geimsa stained. Representative image of (A) cells in culture and (B) erythroid islands with erythroblasts in karyokinesis (arrows) are presented (scale bar 25 μm).
(PDF)

S5 Fig Gene expression profile of CGS derived macrophages versus BM cells. The expression profiles for ferroportin, DNASE2, CD163, ICAM-4 and ITGAM were determined by real-time qPCR in CGS expanded CB derived CD206+ macrophages at day 14 of culture and unmanipulated CD14+ cells derived from healthy BM donors. Expression levels were normalized to the housekeeping gene GAPDH and are reported as the Log2 fold change. Individual data points are from six independent healthy BM donors and three pooled CB donors from two independent CGS culture.
(PDF)

S6 Fig Bone marrow stromal cell conditioned medium change the morphology of monocytes. CD14+ monocytes were isolated by immunomagnetic separation from unmanipulated cord blood mononuclear cells. Monocytes were cultured in IMDM/FBS (No CM), supplemented with HS27a and HS5 CM for three days. Cells were harvested, cytospun and Wright-Geimsa stained. Microscopic study revealed a change in the morphology of the monocytes in response to the conditioned medium (scale bar 25 μm).
(PDF)

S7 Fig Recombinant human VCAM-1 enhance CGS induced erythroid differentiation of CB CD34+ cells. Cord blood derived CD34+ cells were transduced with Lentiviral vector encoding the CGS and expanded in the presence of 100 nM AP20187 supplemented with rhVCAM-1 or HS27a conditioned medium. Fold change in CD35a+ erythroid cells at day 7 and 14 relative to day 3 is presented. CB CD34+ cells were derived from two independent cord blood donors and average of two experiments is shown.
(PDF)

S1 FileMaterial and Methods. RT-qPCR.RNA was extracted from CD14+ BM derived monocytes and erythroid island associated CD206+ macrophages (sorted at day 14 CGS expansion culture) by Direct-Zol kit (Zymo Research). cDNA was synthesized using Maxima H minus first strand cDNA synthesis kit (ThermoFisher), and quantified by PowerUp SYBR Green Master Mix (ThermoFisher). Cycling conditions were 50°C for 2 min, 95°C for 5 min, and 49 cycles of 95°C for 15 sec, 60°C for 30 sec. Data are represented as Log2 delta delta Ct values after normalization to *GAPDH* mRNA levels. Primers used in this experiment are listed below.
$$\begin{array}{ll} \text{GAPDH} & \text{F}:\text{ AAGGCTGGGGCTCATTTGC}\\ & \text{R}:\text{ GAGGCATTGCTGATGATCTTG}\\ \text{CD}163 & \text{F}:\text{ AGCGGCTTGCAGTTTCCTC}\\ & \text{R}:\text{ GGAATTTTCTGAGGAATTCATTAGG}\\ \text{ICAM}4 & \text{F}:\text{ GATCACCGCCTACAAACCG}\\ & \text{R}:\text{ GGGAACACCTGCGTCACG}\\ \text{DNASE}2 & \text{F}:\text{ CAGCCAGCTCGCCTTCC}\\ & \text{R}:\text{ CCCCCATCGTGGTCAAGG}\\ \text{ITGAM} & \text{F}:\text{ ACAGCCTTGTTTCCCTTTGAG}\\ & \text{R}:\text{ ATTTCTCACAGTCACTGTCACG}\\ \text{SLC}40\text{A}1 & \text{F}:\text{GACTTAAAGTGGCCCAGACC}\\ & \text{R}:\text{GCAGGAAGTGAGAACCCATC} \end{array}$$(PDF)
