# Supplementary figures and images for: Human Cord Blood and Bone Marrow CD34+ Cells Generate Macrophages That Support Erythroid Islands
Source: PLoS One. 2017 Jan 30;12(1):e0171096. doi: 10.1371/journal.pone.0171096 (PMC5279789; doi:10.1371/journal.pone.0171096)

SFig 1

**A**

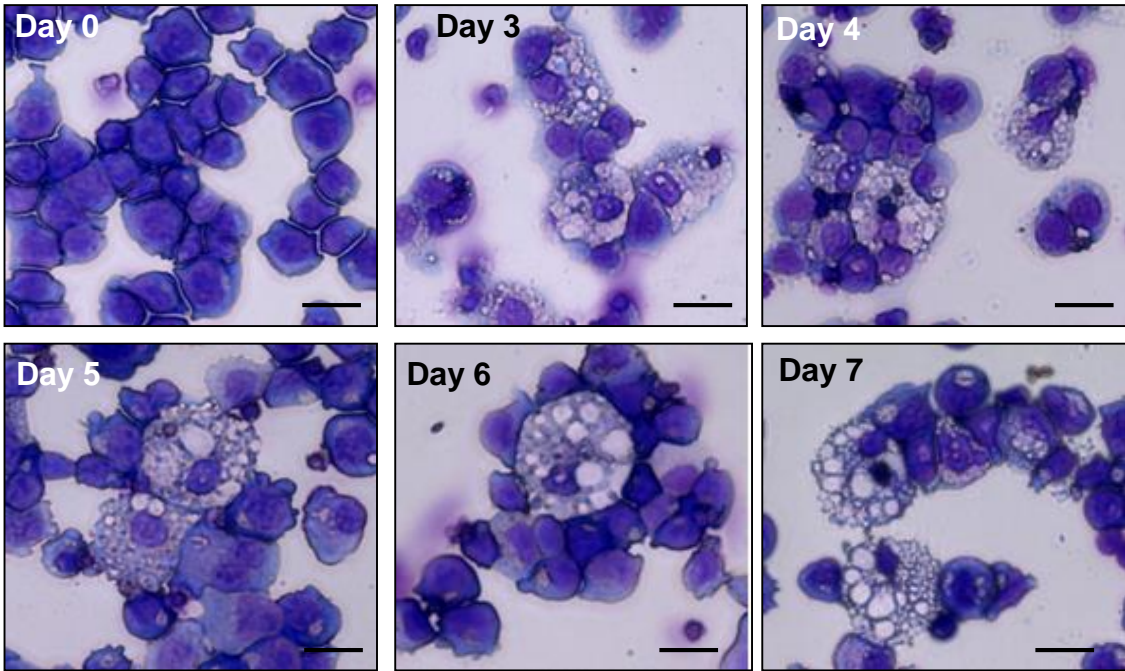

**B**

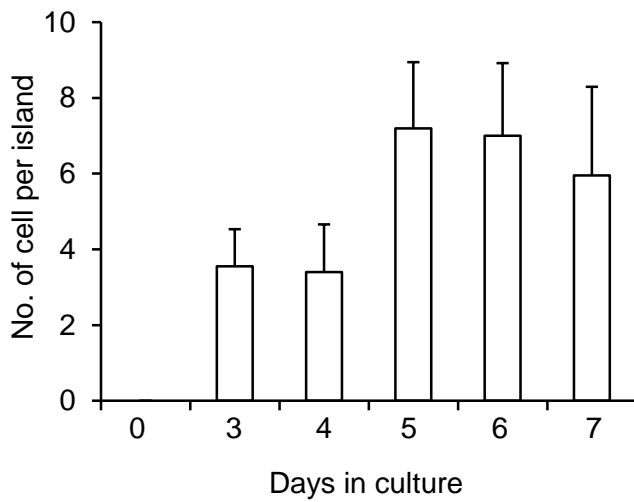

n=20 random islands

Supplement: S1 Fig — Cord blood CD34+ cells were transduced with Lentiviral vector encoding the CGS and plated in IMDM/FBS supplemented with 100 nM AP20187. Cells were harvested at the indicated time points, cytospun and stained with Wright- Giemsa. (A) Representative images of erythroblast and erythroid islands in culture and (B) Average number of cells surrounding a central macrophage. The mean number of erythroblast around a macrophage in 20 randomly selected islands is presented. (scale bar 25 μm). (PDF) [file pone.0171096.s001.pdf]

## Supplementary Figure 2

Phenotype of sorted macrophages (CD14+)

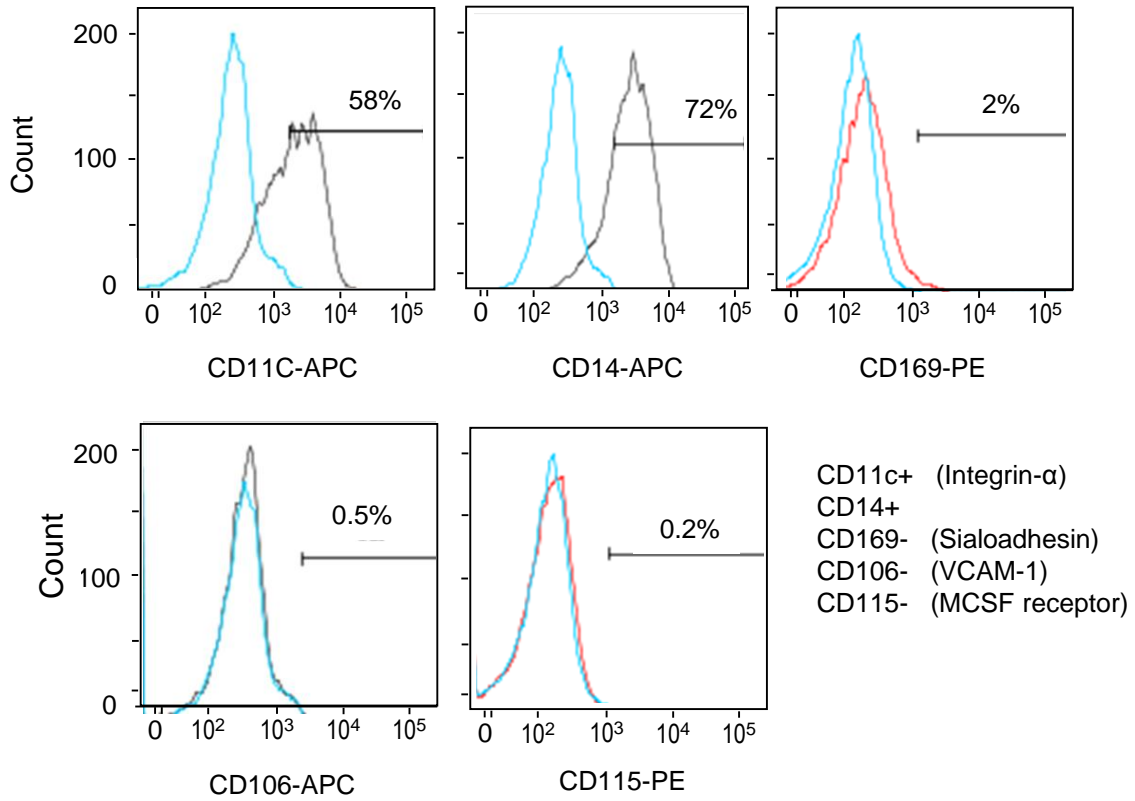

Supplement: S2 Fig — Cord blood CD34+ cells were transduced with Lentiviral vector encoding the CGS and plated in IMDM/FBS supplemented with 100 nM AP20187. At day 14 of culture cells were harvested stained with CD206 antibody and flow sorted. Sorted macrophages were stained with the indicated cell surface markers and flow analyzed, representative flow cytometric histograms are presented. (PDF) [file pone.0171096.s002.pdf]

# SFig3

Colony 1

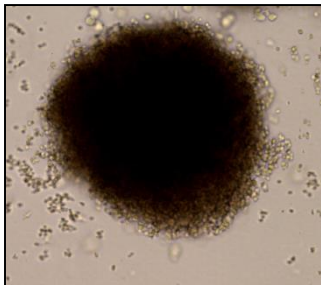

Colony 2

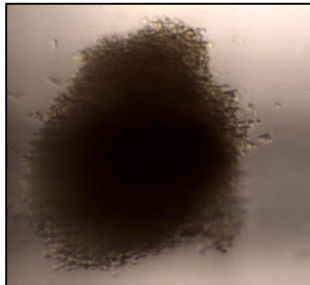

Colony 3

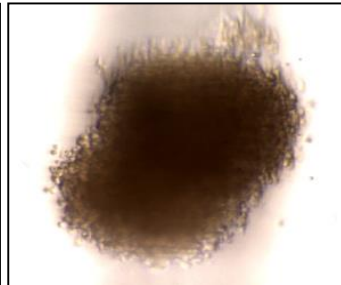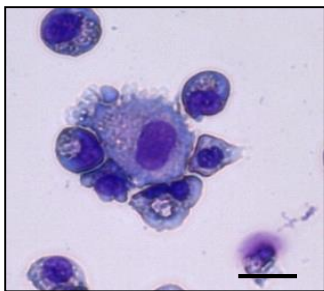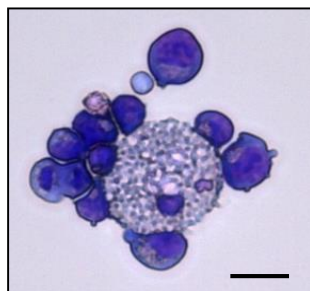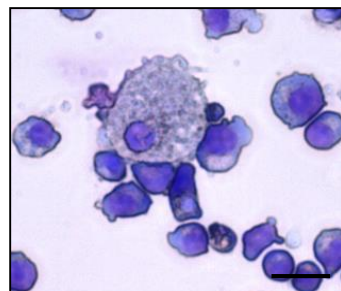

Colony 4

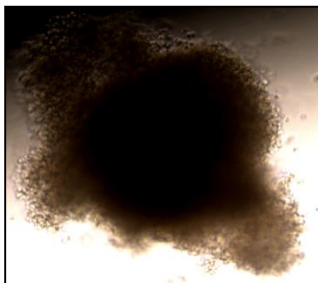

Colony 5

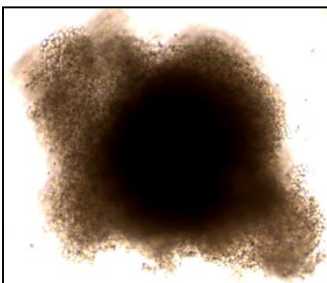

Colony 6

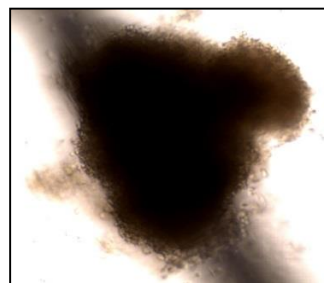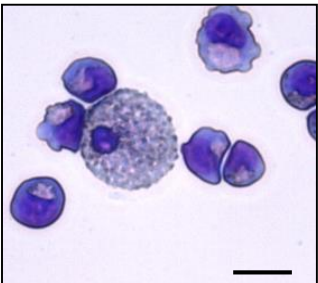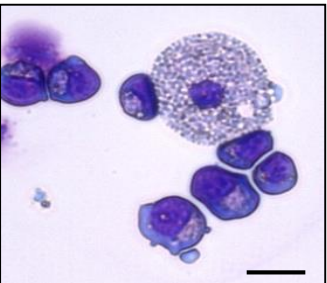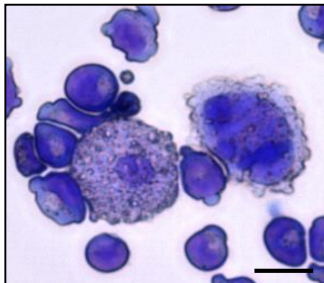

Supplement: S3 Fig — Cord blood CD34+ cells were transduced with Lentiviral vector encoding the CGS and plated in a cytokine free MethoCult semi solid medium supplemented with 100 nM AP20187. At day 14, red colonies were manually picked and Wright-Giemsa stained. Representative images of individual colonies and the corresponding central macrophage from each colony are presented (n = 20 colonies, scale bar 25 μm). (PDF) [file pone.0171096.s003.pdf]

Supplementary Figure 4

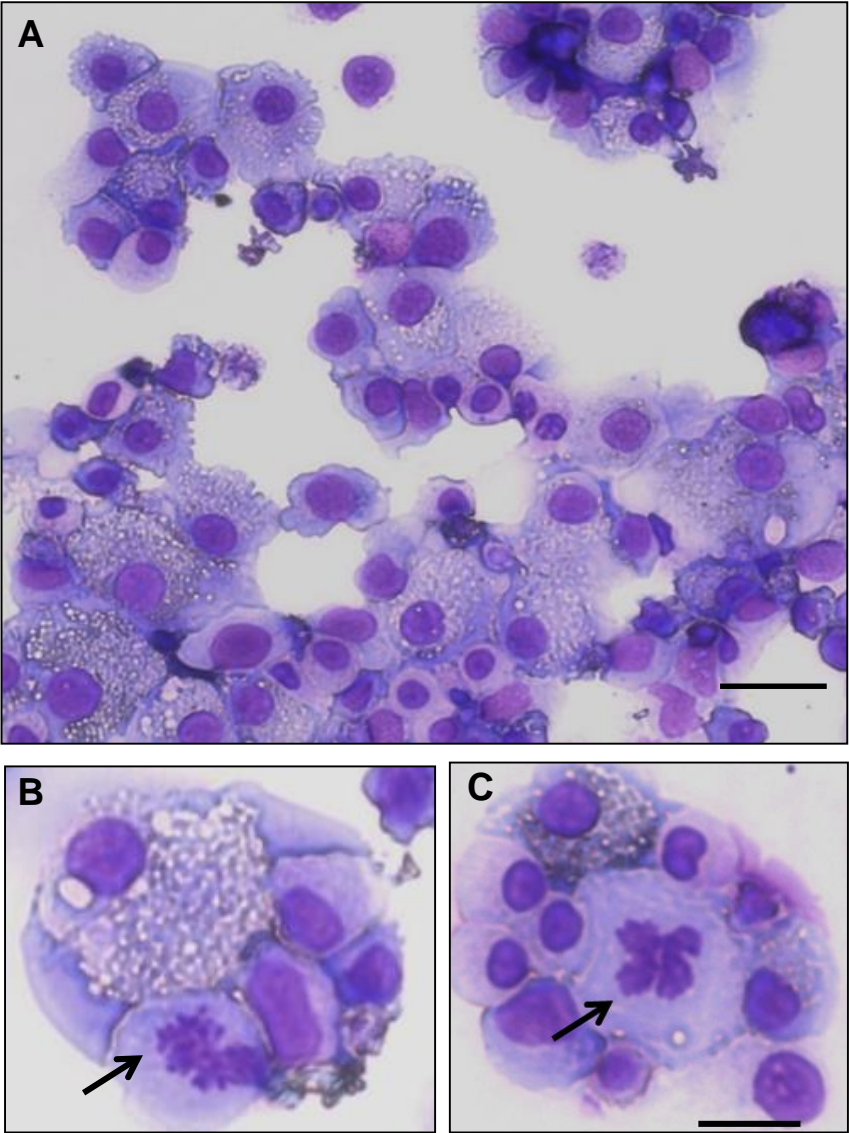

Supplement: S4 Fig — Bone marrow CD34+ cells were transduced with Lentiviral vector encoding the CGS, and plated in a cytokine free MethoCult semi solid medium supplemented with 100 nM AP20187. After collection of time lapse images cells were harvested, cytospun and Wright-Geimsa stained. Representative image of (A) cells in culture and (B) erythroid islands with erythroblasts in karyokinesis (arrows) are presented (scale bar 25 μm). (PDF) [file pone.0171096.s004.pdf]

Sfig 5

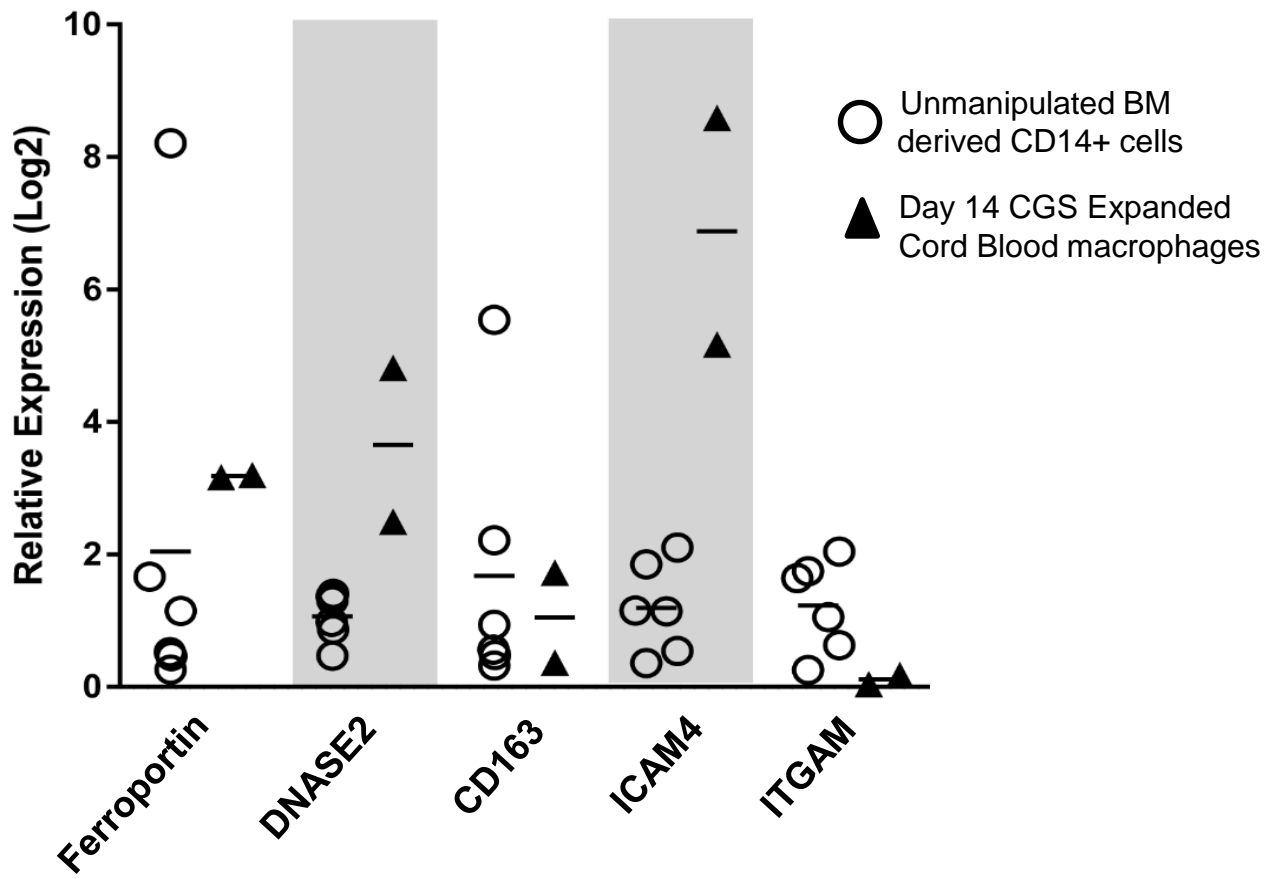

Supplement: S5 Fig — The expression profiles for ferroportin, DNASE2, CD163, ICAM-4 and ITGAM were determined by real-time qPCR in CGS expanded CB derived CD206+ macrophages at day 14 of culture and unmanipulated CD14+ cells derived from healthy BM donors. Expression levels were normalized to the housekeeping gene GAPDH and are reported as the Log2 fold change. Individual data points are from six independent healthy BM donors and three pooled CB donors from two independent CGS culture. (PDF) [file pone.0171096.s005.pdf]

SFig6

CD14+ monocytes  
No CM

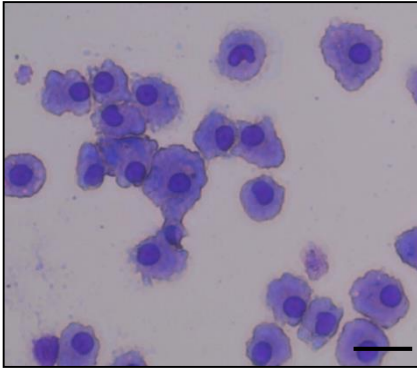

CD14+ monocytes  
+ HS27a CM

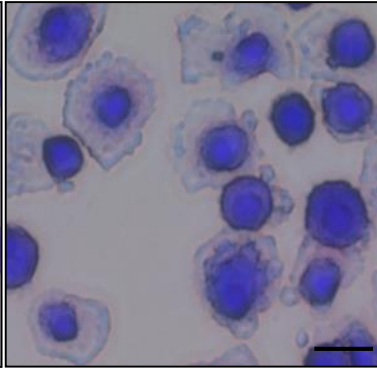

CD14+ monocytes  
+ HS5 CM

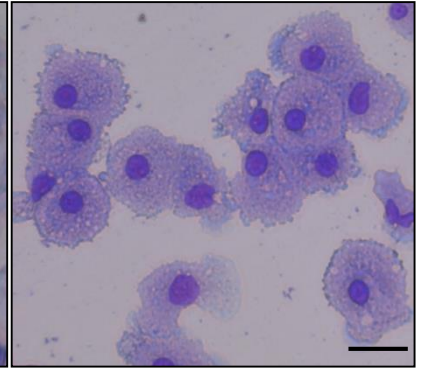

Supplement: S6 Fig — CD14+ monocytes were isolated by immunomagnetic separation from unmanipulated cord blood mononuclear cells. Monocytes were cultured in IMDM/FBS (No CM), supplemented with HS27a and HS5 CM for three days. Cells were harvested, cytospun and Wright-Geimsa stained. Microscopic study revealed a change in the morphology of the monocytes in response to the conditioned medium (scale bar 25 μm). (PDF) [file pone.0171096.s006.pdf]

SFig7

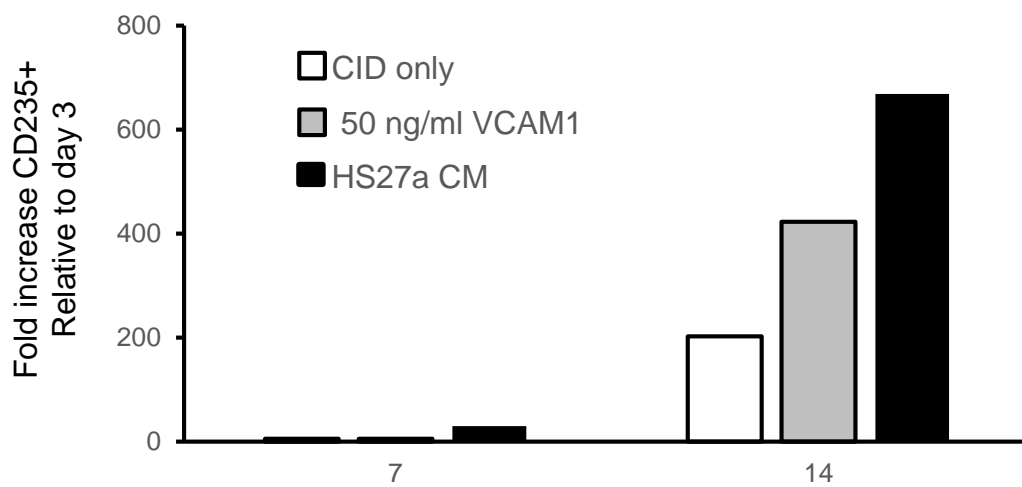

Supplement: S7 Fig — Cord blood derived CD34+ cells were transduced with Lentiviral vector encoding the CGS and expanded in the presence of 100 nM AP20187 supplemented with rhVCAM-1 or HS27a conditioned medium. Fold change in CD35a+ erythroid cells at day 7 and 14 relative to day 3 is presented. CB CD34+ cells were derived from two independent cord blood donors and average of two experiments is shown. (PDF) [file pone.0171096.s007.pdf]
