# Supplementary material for: Human Cord Blood and Bone Marrow CD34+ Cells Generate Macrophages That Support Erythroid Islands
Source: PLoS One. 2017 Jan 30;12(1):e0171096. doi: 10.1371/journal.pone.0171096 (PMC5279789; doi:10.1371/journal.pone.0171096)
Supplement: S1 File — RNA was extracted from CD14+ BM derived monocytes and erythroid island associated CD206+ macrophages (sorted at day 14 CGS expansion culture) by Direct-Zol kit (Zymo Research). cDNA was synthesized using Maxima H minus first strand cDNA synthesis kit (ThermoFisher), and quantified by PowerUp SYBR Green Master Mix (ThermoFisher). Cycling conditions were 50°C for 2 min, 95°C for 5 min, and 49 cycles of 95°C for 15 sec, 60°C for 30 sec. Data are represented as Log2 delta delta Ct values after normalization to GAPDH mRNA levels. Primers used in this experiment are listed below. GAPDHF: AAGGCTGGGGCTCATTTGCR: GAGGCATTGCTGATGATCTTGCD163F: AGCGGCTTGCAGTTTCCTCR: GGAATTTTCTGAGGAATTCATTAGGICAM4F: GATCACCGCCTACAAACCGR: GGGAACACCTGCGTCACGDNASE2F: CAGCCAGCTCGCCTTCCR: CCCCCATCGTGGTCAAGGITGAMF: ACAGCCTTGTTTCCCTTTGAGR: ATTTCTCACAGTCACTGTCACGSLC40A1F:GACTTAAAGTGGCCCAGACCR:GCAGGAAGTGAGAACCCATC (PDF) [file pone.0171096.s008.pdf]

S1 File. Material and Methods

RT-qPCR. RNA was extracted from CD14+ BM derived monocytes and erythroid island associated CD206+ macrophages (sorted at day 14 CGS expansion culture) by Direct-Zol kit (Zymo Research). cDNA was synthesized using Maxima H minus first strand cDNA synthesis kit (ThermoFisher), and quantified by PowerUp SYBR Green Master Mix (ThermoFisher). Cycling conditions were 50°C for 2 min, 95°C for 5 min, and 49 cycles of 95°C for 15 sec, 60°C for 30 sec. Data are represented as Log2 delta delta Ct values after normalization to *GAPDH* mRNA levels. Primers used in this experiment are listed below.

GAPDH	F: AAGGCTGGGGCTCATTTGC
	R: GAGGCATTGCTGATGATCTTG
CD163	F: AGCGGCTTGCAGTTTCCTC
	R: GGAATTTTCTGAGGAATTCATTAGG
ICAM4	F: GATCACCGCCTACAAACCG
	R: GGGAACACCTGCGTCACG
DNASE2	F: CAGCCAGCTCGCCTTCC
	R: CCCCCATCGTGGTCAAGG
ITGAM	F: ACAGCCTTGTTTCCCTTTGAG
	R: ATTTCTCACAGTCACTGTCACG
SLC40A1	F: GACTTAAAGTGGCCCAGACC
	R: GCAGGAAGTGAGAACCCATC
